# Process-Oriented Review of Bacterial Quorum Quenching for Membrane Biofouling Mitigation in Membrane Bioreactors (MBRs)

**DOI:** 10.3390/membranes6040052

**Published:** 2016-12-13

**Authors:** Naila Bouayed, Nicolas Dietrich, Christine Lafforgue, Chung-Hak Lee, Christelle Guigui

**Affiliations:** 1 LISBP—Laboratoire d’Ingénierie des Systèmes Biologiques et des Procédés), CNRS—Centre National de la Recherche Scientifique), INRA—Institut National de la Recherche Agronomique), INSA—Institut National des Sciences Appliquées), Université de Toulouse, Toulouse 31077, France; dietrich@insa-toulouse.fr (N.D.); lafforgu@insa-toulouse.fr (C.L.); guigui@insa-toulouse.fr (C.G.); 2 School of Chemical and Biological Engineering, Seoul National University, Seoul 08826, Korea; leech@snu.ac.kr

**Keywords:** membrane bioreactors, biofouling, biofilm, Quorum Sensing, Quorum Quenching, acyl-homoserine lactones, acylase, lactonase, extracellular polymeric substances (EPS)

## Abstract

Quorum Quenching (QQ) has been developed over the last few years to overcome practical issues related to membrane biofouling, which is currently the major difficulty thwarting the extensive development of membrane bioreactors (MBRs). QQ is the disruption of Quorum Sensing (QS), cell-to-cell communication enabling the bacteria to harmonize their behavior. The production of biofilm, which is recognized as a major part of the biocake formed on a membrane surface, and which leads to biofouling, has been found to be one of the bacterial behaviors controlled by QS. Since the enzymatic disruption of QS was reported to be efficient as a membrane biofouling mitigation technique in MBRs, the application of QQ to lab-scale MBRs has been the subject of much research using different approaches under different operating conditions. This paper gives an overview of the effectiveness of QQ in mitigating membrane biofouling in MBRs. It is based on the results of previous studies, using two microbial strains, *Rhodococcus* sp. BH4 and *Pseudomonas* sp. 1A1. The effect of bacterial QQ on the physical phenomena of the MBR process is analyzed, adopting an original multi-scale approach. Finally, the potential influence of the MBR operating conditions on QQ effectiveness is discussed.

## 1. Introduction

Membrane bioreactors (MBRs) are considered as the most effective technology in advanced wastewater treatment (WWT). In comparison to other conventional WWT processes, MBRs provide excellent water quality with a smaller footprint, offering a great potential for water reuse. However, membrane fouling remains a major obstacle that still tends to hold back the wide application of MBRs. Membrane fouling is a term that encompasses all the phenomena inducing the deterioration of membrane performance by a severe loss of permeability, which further results in higher energy consumption to maintain the process productivity and a heightened need to clean or replace the membrane, which finally leads to a substantial increase in operating costs.

Membrane fouling is partly attributed to biofouling, which is defined as the complex combination of several mechanisms: deposition and accumulation of biosolids from the mixed liquor (microbial cells, microbial flocs), microbial growth on the membrane, pore clogging by microorganisms and adsorption of secreted microbial products, which results in the formation of a complex biocake on the membrane. A major part of the biocake is composed of biofilm, which refers to the bacterial tendency to grow as a confined population forming cell clusters embedded in a self-produced slimy matrix composed of extracellular polymeric substances (EPS) [1,2]. In the last decade, several studies have focused on demonstrating the involvement of Quorum Sensing (QS) in biofouling, as reviewed by Lade et al. [3,4] and Siddiqui et al. [5]. QS is defined as cell-to-cell communication enabling the bacteria to harmonize their behavior and to function as a coordinated social cluster. QS, which was first described by Fuqua et al. [6] as the bacterial capability to express certain phenotypes only when a certain cell density threshold is reached, is actually based on the ability of bacteria to produce, release, assess and respond to chemical signals called autoinducers. Three different types of autoinducers have been identified to date: *N*-acyl-l-homoserine lactones (acyl-HSLs or AHLs) for Gram-negative communication, autoinducing peptides (AIPs) for Gram-positive bacteria and furanosyl borate diesters known as AI-2 signals for interspecies communication.

**Figure 1:**
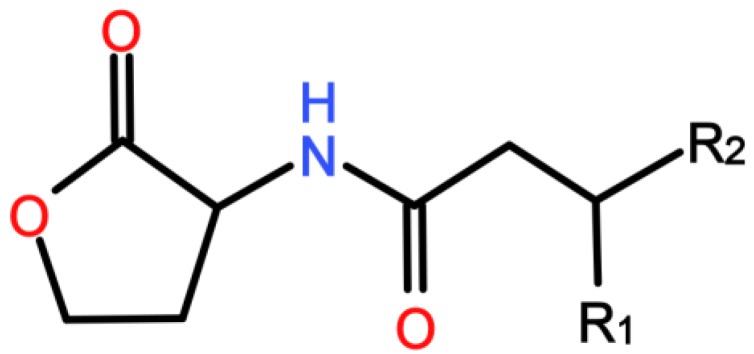
Structure of an *N*-acyl-l-homoserine lactones (AHL) molecule (R1 can be an oxo or a hydroxyl group; R2 can be a carbon chain from C1 to C15).

In MBRs, the Gram-negative proteobacteria phylum was demonstrated to be the most abundant among the myriad MBR-living bacteria [7,8,9]. Thus, the prevalent bacterial communication in MBRs is believed to be Gram-negative QS which is ensured through AHLs as autoinducers. These molecules are composed of a lactone ring and an acylated chain containing four to 18carbons as reviewed by Lade et al. [3] (Figure 1).

AHL-mediated QS in Gram-negative bacteria is based on the biosynthesis of AHLs in the intracellular compartment, then on their accumulation both in the intracellular and the extracellular environment because of their small molecular weights. At high cell density, the extracellular concentration of AHLs increases until it reaches a threshold, beyond which bacteria can sense them and then activate the transcription of certain target genes (Figure S1) [10,11,12,13,14]. For more details on AHL-mediated QS for Gram-negative bacterial communication, the reader is invited to refer to the Supporting Information.

AHL-mediated QS has been proved to be involved in biofilm formation in various ways for a number of bacterial species [15,16,17,18,19,20,21,22,23]. A few studies on biofouling control in MBRs have focused on the potential relationship between AHLs and biofilm on the membrane. Yeon et al. [24] were the first to provide evidence of the occurrence of AHL-mediated QS in MBRs by proving the presence of different AHLs in the biocake formed on the membrane surface. This result has been confirmed [25,26,27,28,29,30] and then taken to the next step with the determination of a correlation between the AHL amount and the transmembrane pressure (TMP) level [24,29]. Hence, the occurrence of AHL-mediated QS has not only been highlighted but has also been demonstrated to be closely related to biofouling in MBRs.

With this observation, a novel biofouling mitigation strategy based on QS, i.e., Quorum Quenching (QQ), was then developed. As its name implies, QQ refers to any mechanism that can effectively disrupt QS communication [31]. In theory, QS inhibition can be achieved by targeting either the generation of AHL, the AHL molecule itself, or the AHL reception, as reviewed by Rasmussen and Givskov [32]. In the particular case of biofouling in MBRs, QQ through destruction of the AHL molecules has been the most investigated mechanism. In essence, QQ can be achieved using several QS inhibitors (QSIs), among which natural compounds, such as piper betle extract (PBE) [29,33] and AHL-degrading enzymes or bacteria, have been preferred over synthetic QSIs for application to MBRs. To date, the degradation of AHLs is the most appreciated and the most commonly applied method for biofouling mitigation in MBRs, and this has been achieved by using either a purified QQ enzyme (porcine kidney acylase I [24,34,35,36,37]) or bacteria that produce a QQ enzyme (QQ bacteria). The former technique is referred to as “enzymatic QQ” in opposition to the latter, which is known as “bacterial QQ”.

Recently, three interesting reviews have given overviews of the QS and QQ mechanisms, together with summaries of all the QSIs and all the QS-based ways to mitigate membrane biofouling in general [3,4,5]). However, these reviews do not plainly explain biofouling in MBRs: the links between QQ and the physical phenomena of the MBR process are not explored in depth, and the recent trends in QS-based biofouling mitigation are not fully supported by numerical data.

In the present review, special emphasis will be placed on the application of bacterial QQ to mitigate membrane biofouling in MBRs for WWT. To date, the literature reports that two major QQ bacteria have a capacity to mitigate biofouling in MBRs: *Rhodococcus* sp. BH4 [28] and *Pseudomonas* sp. 1A1 [38]. These two bacteria appear to have different modes of action to mitigate membrane biofouling, which are mostly attributable to the nature and the localization of the QQ enzymes they produce. Thus, we propose to discuss the relationship between the bacterial QQ and the MBR process here, taking the two different points of view offered by the confrontation of these two QQ bacteria. In that way, for each of these strains, the mode of action will be explained by giving details about the nature and the localization of the QQ enzyme it produces. The QQ effectiveness will be further discussed by analyzing the effects of bacterial QQ on the physical phenomena related to biofouling in MBRs at both the macroscopic and microscopic scales. Then, the effects of the MBR operating conditions on QQ effectiveness will be examined to try to highlight potentially optimal conditions for bacterial QQ application in MBRs. Finally, some concluding remarks and perspectives will be presented in the last part as directions for future research.

## 2. History of Bacterial QQ

The application of bacterial QQ by means of QQ-enzyme-producing bacteria was developed to overcome practical barriers related to the use of a purified enzyme, such as the purification costs and the loss of the enzyme activity. Oh et al. [28] were the first to experiment bacterial QQ in a lab-scale MBR. For that purpose, a batch-type MBR was supplemented with a genetically modified *E. coli* strain harboring the aiiA gene, coding for the production of a QQ enzyme. As a result, 30% of reduction in the TMP level was observed, and the time for the TMP to reach 25 kPa was extended by approximately 40%. The use of this recombinant *E. coli* has further led to similar results in a continuous mode, demonstrating the potential of bacterial QQ to mitigate biofouling in lab-scale MBRs. However, the use of such a recombinant strain is hardly conceivable because of its very poor chances of survival in a real MBR but also because of the need to introduce antibiotics to maintain its QQ activity. Considering the significant number of natural QQ bacteria that have been identified to date (reviewed by Czajkowski and Jafra [39] and Lade et al. [3]), the isolation of an indigenous QQ bacterium from a real MBR appeared to be the most reasonable method. Thus, *Rhodococcus* sp. BH4 and *Pseudomonas* sp. 1A1 were isolated, characterized, and tested as a QQ bacteria to mitigate membrane biofouling in MBRs.

## 3. Isolation of QQ Bacteria

*Rhodococcus* sp. BH4 is a Gram-positive bacterium that happens to be the first indigenous strain isolated from a real MBR for its QQ potential, using an enrichment culture method as described by Oh et al. [28]. To achieve this, activated sludge (AS) samples or biocake samples were taken from a real MBR to be further inoculated in a minimal medium containing AHLs. After incubation, the culture was transferred to a fresh minimal medium, and the transfer procedure was repeated three times to ensure the isolation of bacteria that could live with AHLs as sole carbon source. Finally, single colonies were isolated on LB agar then separately incubated in a minimal medium. Among the few strains isolated by this method, a strain showing high activity against C8-HSL was identified as a *Rhodococcus* sp. BH4 by 16S rRNA sequence analysis and was finally selected for investigation its QQ potential to reduce biofouling in MBRs. *Pseudomonas* sp. 1A1 is a Gram-negative bacterium that was further isolated from a real MBR using a minimal medium containing AHL as the sole source of carbon, according to the same enrichment method [38].

## 4. Roles of *Rhodococcus* sp. BH4 and *Pseudomonas* sp. 1A1 as QQ Bacteria

In order to identify the AHL-degrading enzyme produced by *Rhodococcus* sp. BH4, a genetic comparison with the known *Rhodococcus erythropolis* W2 was conducted and revealed the presence of the same AHL-lactonase gene [40]. Hence, the QQ activity of *Rhodococcus* sp. BH4 against AHLs is strongly believed to be ensured by lactonase. However, some additional research may be needed, since other strains belonging to the *Rhodococcus* genus have been found to produce other kinds of AHL-degrading enzymes, such as acylases and oxidoreductases [41,42,43].

**Figure 2:**
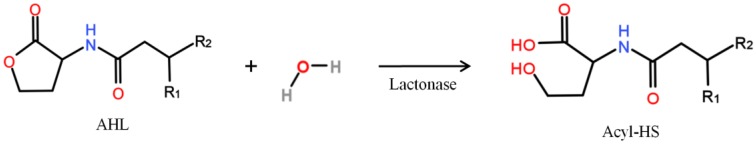
AHL degradation by lactonase produced by *Rhodococcus* sp. BH4.

Lactonase has the capacity to degrade AHLs by opening the lactone ring according to the enzymatic reaction presented in Figure 2. This mode of action suggests that lactonase can theoretically degrade a wide range of AHLs regardless of the acyl chain lengths and the substitutions on the third carbon (R1 group on Figure 2), evoking a non-selective QQ activity. However, it has been reported that lactonase from *Rhodococcus* sp. BH4 has a lower degrading activity against AHLs with an additional oxo group on the third carbon, compared to the same AHLs with no substitutions [44]. In addition, for AHLs with an oxo group, the lactonase activity increases with the length of the acyl chain, which finally suggests a greater affinity for some AHLs than for others [40,44]. It has been demonstrated that the lactonase activity may be impaired under acidic pH or at high temperature [44]. In addition, the latter study has revealed that the catalytic activity of some lactonases can be totally inhibited by the presence of certain metal ions such as Cu^2+^ and Ag^+^ at 0.2 mM [45]. Thus, when using *Rhodococcus* sp. BH4 as a lactonase-producing strain for biofouling mitigation in an MBR, it can be of great interest to check that the amounts of Cu^2+^ and Ag^+^ in the MBR are below the inhibition limit of 0.2 mM that has been reported [44]. Finally, a potential drawback of using lactonase from *Rhodococcus* sp. BH4 as a QQ enzyme is that the AHL degradation reaction is reversible under acidic pH. However, the rebinding can be avoided by a chemical modification of the opened ring (substitution or reduction) [32].

For *Pseudomonas* sp. 1A1, a genetic analysis revealed the presence of an AHL-acylase homologue gene, which implies that the QQ activity of this strain towards AHLs is most likely ensured by an acylase [38]. This finding is consistent with a previous study investigating the QQ enzyme produced by another *Pseudomonas* strain (PAO1) [46].

**Figure 3:**
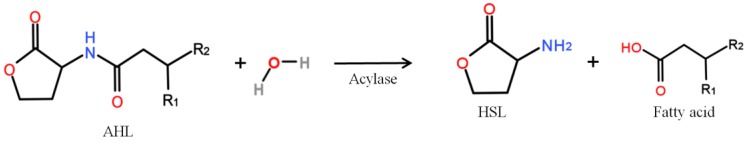
AHL degradation by acylase produced by *Pseudomonas* sp. 1A1.

Acylase is known to degrade AHLs by hydrolyzing the amine bond between the acyl chain and the lactone ring to produce homoserine lactones and fatty acids (Figure 3). Concerning the enzymatic activity of acylase from *Pseudomonas* sp. 1A1, it has been proved that the longer the acyl side chain is, the greater is the degradation rate, for both substituted and unsubstituted AHLs ranging from C6 to C12 [38]. Similar results have been obtained when characterizing an acylase from *Anabaena* sp. PCC7120, which confirms that the acylase activity depends on the side chain length [47]. Furthermore, it is highly probable that the activity of acylase from *Pseudomonas* sp. 1A1 is dependent on pH and temperature, as has been demonstrated for a purified acylase, porcine kidney acylase [48].

## 5. Methods for Entrapping QQ Bacteria in MBRs

After the identification of these two QQ bacteria, their implementation in MBRs was carried out to assess their potential to mitigate biofouling by interfering with AHL signal molecules. For that purpose, different entrapping methods were used, in order to avoid loss of cells with the withdrawal of excess sludge from the MBR, as well as to protect them against the attack of other microorganisms cohabiting in the MBR, QQ bacteria were added using three entrapping methods: (i) microbial vessels for entrapping *Rhodococcus* sp. BH4 and *Pseudomonas* sp. 1A1 (ii) sodium alginate beads and (iii) a Rotating Microbial Carrier Frame (RMCF), for *Rhodococcus* sp. BH4.

**Figure 4:**
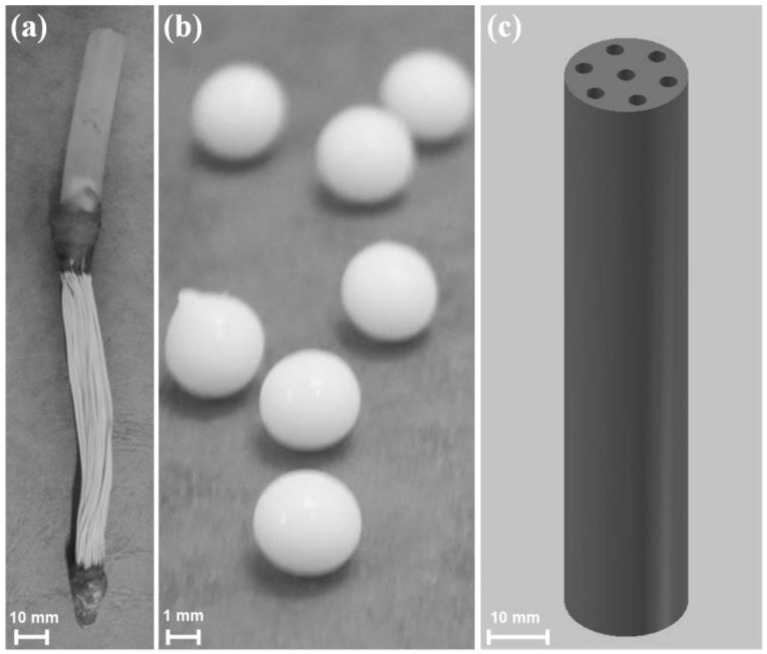
(**a**) Polyethylene (PE) microbial vessel; (**b**) sodium alginate beads for *Rhodococcus* sp. BH4 entrapment and (**c**) ceramic microbial vessel (CMV) for *Pseudomonas* sp. 1A1 entrapment.

(i) Microbial vessels were designed to maintain the QQ bacteria immobilized using a porous material permitting the free diffusion of AHLs and nutrients through the vessel. For example, Oh et al. [28] used a module composed of hollow, 10-cm long polyethylene (PE), fibers to encapsulate the *Rhodococcus* sp. BH4 cells (Figure 4a). The module was then submerged and held in a fixed place in the MBR. The initial amounts of *Rhodococcus* sp. BH4 cells inserted in microbial vessels ranged from approximately 130 to 450 mg/L of the total working volume [28,40,49]. The volume of the vessel itself represented less than 0.08% of the MBR volume. For *Pseudomonas* sp. 1A1, two types of materials were used for these vessels: PE and ceramic. As for *Rhodococcus* sp. BH4, Cheong et al. [38] made PE vessels using microporous HF (0.4 µm). They also designed a ceramic microbial vessel (CMV) consisting of a monolithic microporous module (0.45 µm) with a total length of 10 cm and composed of several lumens into which the cells were injected using a sterile syringe (Figure 4c). The initial quantities of *Pseudomonas* sp. 1A1 cells tested varied from approximately 200 to 700 mg 1A1/L of the total working volume.
(ii) Sodium alginate beads were only used for entrapping *Rhodococcus* sp. BH4. They were prepared by dripping a mixture of a *Rhodococcus* sp. BH4 suspension and a sodium alginate solution through a nozzle into a CaCl_2_ solution to obtain spherical Cell Entrapping Beads (CEBs) as described by Kim et al. [25] (Figure 4b). In that case, the amount of *Rhodococcus* sp. BH4 entrapped in the beads was around 6 mg BH4/g sodium alginate. Kim et al. [50] further developed macrocapsules by coating the CEBs with a microporous polymer layer for better resistance to harsh conditions resulting from the use of real WW. Recently, Lee et al. [26] developed QQ beads made of a mixture of polyvinyl alcohol and sodium alginate to reinforce their stability in real WW. The beads were then inserted into the MBR where they could move freely.
(iii) Recently, an RMCF has been developed as a new entrapping technology for *Rhodococcus* sp. BH4, using a polycarbonate frame and four cubbyholes covered with a polyvinylidene fluoride (PVDF) microfiltration membrane, and packed with a *Rhodococcus* sp. BH4 suspension using a syringe. The RMCF was then set into the MBR similarly to a mechanical stirring device [51].

## 6. Localization of the Activity of the QQ Bacteria in the MBR

**Figure 5:**
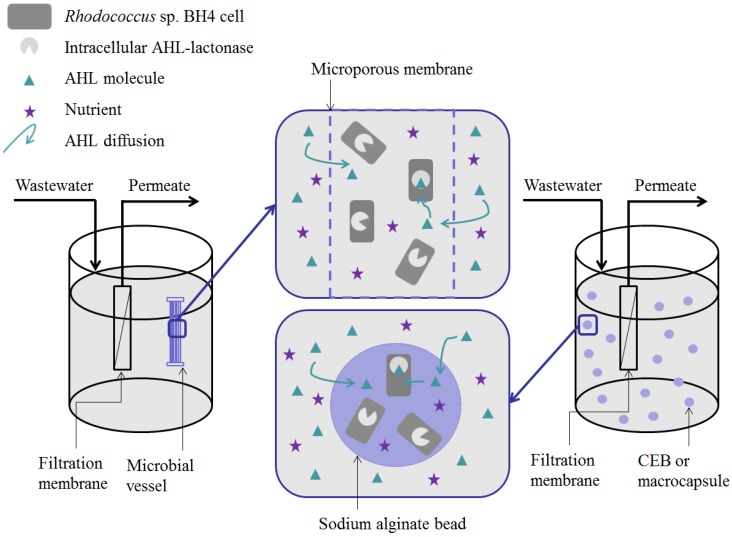
Localization of the Quorum Quenching (QQ) activity of *Rhodococcus* sp. BH4 entrapped in a microbial vessel or sodium alginate beads in a lab-scale membrane bioreactor (MBR).

Measuring the enzymatic activities of the supernatant and the pellets from the same *Rhodococcus* sp. BH4 suspension sample has revealed that the QQ activity of *Rhodococcus* sp. BH4 is based on the production of an intracellular AHL-lactonase (endo-enzyme), which indicates that the enzymatic degradation of AHLs takes place inside the *Rhodococcus* sp. BH4 cells [40]. This implies that, when *Rhodococcus* sp. BH4 is implemented in an MBR for membrane biofouling control, the AHLs from either the mixed liquor or the biocake on the membrane are transported by convection towards the entrapping element, diffuse through this porous element towards the *Rhodococcus* sp. BH4 cells and then through the cell membrane to be hydrolyzed in their intracellular compartment (Figure 5). When the microbial vessel is used as an entrapping element, the *Rhodococcus* sp. BH4 cells are held in a fixed place in the MBR and the convective forces created by the aeration and the suction are expected to drive the AHLs towards the immobilized *Rhodococcus* sp. BH4 cells. The latest technology for encapsulating the QQ bacteria (RMCF) is not represented in Figure 5 but it is noteworthy that the rotation motion of this module is believed to prevent the sedimentation of cells in the module and to generate additional shear forces in the MBR, which may promote AHL transport. Finally, in the case of beads, it can be assumed that the free movement of the beads in the MBR enhances the probability that AHLs will encounter the *Rhodococcus* sp. BH4 cells. Nevertheless, the complex structure of the sodium alginate core could slow down the diffusion of AHLs through the beads. Therefore, the localization of the QQ activity is key information that can significantly impact the choice of one of these entrapping techniques. In the following, the possible impact of the different entrapping techniques on the effectiveness of QQ to mitigate biofouling will be discussed.

**Figure 6:**
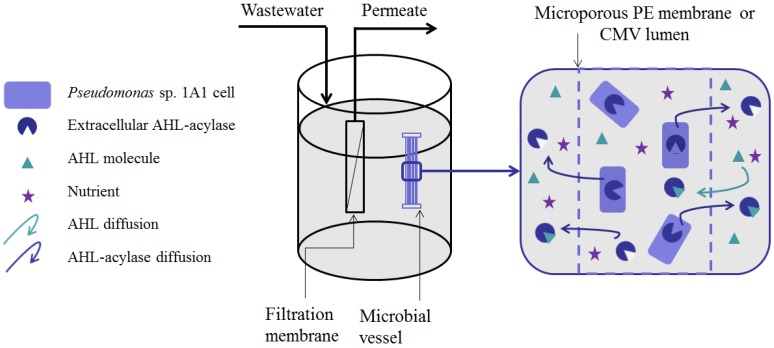
Localization of the QQ activity of *Pseudomonas* sp. 1A1 entrapped in a microbial vessel in a lab-scale MBR.

Unlike lactonase from *Rhodococcus* sp. BH4, acylase produced by *Pseudomonas* sp. 1A1 has been proved to be an exo-enzyme having extracellular activity [38]. This finding has been further reinforced by the fact that acylases released from *Pseudomonas* sp. 1A1 continued to accumulate in the extracellular environment even when the concentration of dead cells started to increase. Thus, the acylase produced by *Pseudomonas* sp. 1A1 is believed to spread freely out of the cells to bind to and hydrolyze AHLs everywhere in the reactor or on the membrane surface as well as inside or outside the entrapping device (Figure 6).

## 7. Performance of the Bacterial QQ-MBR

In order to assess the *Rhodococcus* sp. BH4 capacity to mitigate biofouling in MBRs through a QQ activity, entrapped cells were introduced in different lab-scale MBRs (working volume < 5 L), a semi-pilot MBR (working volume of 35 L) and pilot-scale MBRs (working volume ≥ 80 L) operated in a continuous mode. The MBRs were inoculated with AS from WWT plants, and hollow fibers (HF) or Flat Sheets (FS) were used to ensure the WW filtration. In parallel to these QQ MBRs, control MBRs were systematically run using the entrapping elements with no *Rhodococcus* sp. BH4 cells inside (vacant vessel, vacant beads or vacant RMCF) to maintain exactly the same hydrodynamic conditions. Table 1 gathers together all the studies on the effectiveness of *Rhodococcus* sp. BH4 in reducing biofouling in MBRs.

Concerning *Pseudomonas* sp. 1A1, its potential to mitigate biofouling has been assessed and is shown in Table 2. All experimental data were gathered from the operation of a lab-scale control or QQ-MBR (working volume 5 L) running in a continuous mode, in which a filtration membrane module (PVDF HF) was submerged. Various factors were compared between a control-MBR without *Pseudomonas* sp. 1A1 cells in the vessels and a QQ-MBR with the cells.

A wide variability can be observed in the way results are expressed in these studies. Hence, we propose to standardize these results in terms of percentages of the control MBR performance, so as to better highlight the contribution of QQ to biofouling reduction. The calculation used to convert results into percentages is presented in Equation (1).

(1) $$P\%=-\frac{M_{control\;MBR}-M_{QQ\;MBR}}{M_{control\;MBR}}\times 100$$

*P***%** is the standardized result in percentages; M is one of the properties measured to characterize biofouling; the minus sign (−) stands for the reduction of biofouling by QQ.

## 8. Effect of Bacterial QQ on the MBR Performance at Macroscopic Scale

The effect of QQ on the MBR performance at macroscopic scale is discussed here in terms of TMP, characteristics of the mixed liquor, and biodegradation efficiencies.

The TMP was chosen because it is without any doubt the most monitored property in studies of biofouling in MBRs, most likely because it is an excellent indicator of biofouling and it can be easily measured continuously during the MBR operation, providing key information on the biofouling kinetics. For these reasons, all the studies reporting the effectiveness of QQ to mitigate biofouling are at least partly based on the comparison of the TMP profiles. Before giving numerical results, we illustrate the general shape of the TMP profile in MBRs, then the possible modifications resulting from the implementation of bacterial QQ and the information that can be deduced from the comparison of the two shapes.

**Figure 7:**
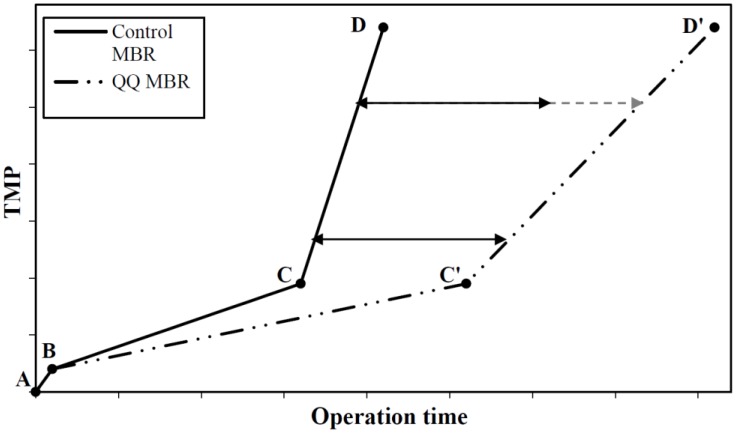
Schematic representation of the TMP profiles obtained in control MBR (solid line) and QQ MBR (dotted line).

Figure 7 presents a schematic illustration of the TMP profiles during MBR operation at constant flux. The fouling phenomenon is now recognized by the MBR community as a three-stage process. Initially, a short-term increase in TMP is observed, from point A to point B, and can probably be attributed to initial pore blocking and the adsorption of solutes from the mixed liquor. Next, a slow long-term rise takes place between points B and C, and is due to the progressive deposition of biosolids (cells and microbial flocs) on the membrane surface and the progressive formation of biofilm. Finally, at point C, called the breaking point, a striking increase, called the TMP jump, indicates a severe loss of permeability [53,54,55]. The occurrence of the TMP jump depends on the operating conditions and is believed to be caused by sudden changes in local flux or in the biocake architecture and EPS composition [55,56,57,58,59,60]. Once point D is reached, the filtration process is stopped and the membrane is either cleaned with a view to reusing it, or changed. The time at which point D is reached is defined as a cycle, and several cycles usually take place during an MBR run.

With the implementation of QQ bacteria to reduce biofouling in MBRs, the second stage of the TMP rise-up is expected to be significantly slowed down from point B to point C’, and the occurrence of the TMP jump (at the breaking point) would thus be delayed (Figure 7). Moreover, the TMP jump from point C’ to point D’ could be expected to be attenuated, indicating that the addition of QQ bacteria could lead to modified biofouling behavior with temporal and spatial variations in both the progressive biofouling stage and the TMP jump stage. Nevertheless, one particular case could be that the TMP jump in the QQ MBR (C’D’ section in Figure 7) was parallel to that in the control MBR (CD section in Figure 7), which could imply that QQ only slows down the progressive biofouling stage with no major changes in the TMP jump stage.

In the studies reported in Table 1 and Table 2, the initial TMP did not exceed 10 kPa (it ranged from approximately 3 to 10 kPa). For all these studies, it was possible to identify the kind of TMP evolution presented in Figure 7. However, the first short-term increase stage (AB section in Figure 7), which takes place relatively quickly, was not clearly identifiable, especially for the studies carried out in the long-term operation.

In order to know which of these stages is the most affected by bacterial QQ, we chose to distinguish between the effect on the progressive biofouling stage (section BC’ in Figure 7) and the effect on the TMP jump (section C’D’ in Figure 7).

Hence, the analysis of the TMP profiles with the latter approach, combined with the characterization of the mixed liquor and the biodegradation efficiencies, are expected to provide information about the direct effects of QQ on the physical phenomena in the MBR at macroscopic scale.

### 8.1. Effect on the Progressive Biofouling Stage

For the assessment of the bacterial QQ effect on the progressive biofouling stage, it may be interesting to take into account the vertical gap between the BC and BC’ sections at the arbitrary time of 1 day (Figure 7). This time has been chosen because it is the time by which the steady progressive biofouling stage was set in all the studies considered in Table 1 and Table 2. This gap would then represent the TMP reduction induced by QQ after 1 day of MBR operation.

After 1 day of operation, a significantly reduced TMP value was noted (references n° 1 to 3 and n° 7 to 12 in Table 1) with more than 50% reduction. On the other hand, for the studies monitoring longer operation times (references n° 4 to 6 and n° 13 and 14 in Table 1), the TMP reduction was less pronounced, with less than 25% reduction. With these results, it appears that *Rhodococcus* sp. BH4-mediated QQ effectively slows down the progressive biofouling stage, which indicates that *Rhodococcus* sp. BH4 probably expresses its QQ activity from the very early phase of the MBR operation (during the first day). However, this effect is less visible for longer operation times.

For *Pseudomonas* sp. 1A1, the TMPs measured in QQ-MBRs were between 15% and 25% lower than that in control-MBRs, after 1 day of operation (references n°1 to 4 in Table 2). These values indicate that *Pseudomonas* sp. 1A1-mediated QQ has a marked effect on the progressive biofouling stage, which means that the *Pseudomonas* sp. 1A1 QQ activity starts relatively early in the MBR operation, slowing down the progressive biofouling stage. Nevertheless, it seems that this effect is less pronounced than that of *Rhodococcus* sp. BH4.

### 8.2. Effect on the TMP Jump

For the evaluation of the *Rhodococcus* sp. BH4-mediated QQ effect on the TMP jump, two criteria have been chosen. The first criterion is the comparison of the times t and t’ corresponding to the times for reaching the breaking points C and C’, respectively (Figure 7), which provide a quantified information about the TMP jump postponement. The second criterion is the comparison of the slopes of the sections CD and C’D’ which were calculated with the points at 25 kPa and 40 kPa that were found to belong to the TMP jump stage for long-term operation.

The analysis of the breaking points according to the first criterion reveals that the time at which the TMP jump occurs is successfully delayed with the implementation of *Rhodococcus* sp. BH4-mediated QQ, by at least 240% (corresponding approximately to a threefold postponement) [49] (references n° 3 to 6 and n° 13 and 14 in Table 1).

For the second criterion, only the long-term MBR operations with running times ranging from 17 to 90 days were taken into account (references n° 5, 6 and 13 in Table 1) since the filtration in shorter operations is usually stopped before (or right after) the breaking point is reached. The time delays to reach the two points of 25 kPa and 40 kPa with QQ were approximately the same for Kim et al. [25,50] (references n° 5 and 13 in Table 1), which refers to similar slopes (around 160 kPa/day for Kim et al. [25] and 25 kPa/day for Kim et al. [50]) for the sections CD and C’D’ (Figure 7). This indicates that *Rhodococcus* sp. BH4-mediated QQ seems to have no effect on the TMP jump stage in these two cases. In contrast, for Lee et al. [26] and Maqbool et al. [27] (references n° 6 and 14 in Table 1), the point of 40 kPa was not actually reached and a less pronounced TMP jump was recorded (data not shown), which indicates a substantial effect of *Rhodococcus* sp. BH4-mediated QQ on the TMP jump.

Studying the effect of *Rhodococcus* sp. BH4-mediated QQ on the TMP jump in addition to its effect on the progressive biofouling stage may give interesting insight into how the QQ effectiveness might evolve over time in the MBR. According to these results, it seems that *Rhodococcus* sp. BH4-mediated QQ tends to be globally more effective in inducing modifications of the TMP profile during the first progressive biofouling stage than during the TMP fast jump stage, which could be attributed to a potential loss of the QQ effectiveness. Another potential explanation could be that another type of QS-controlled biofouling (e.g., AI-2-controlled QS for interspecies communication), against which the *Rhodococcus* sp. BH4 cells have no effect, becomes predominant over the AHL-controlled one. However, further research would be needed to clarify these assumptions. All these results taken together with the number of cycles of the control MBRs compared to the QQ MBRs clearly indicate that the implementation of *Rhodococcus* sp. BH4-mediated QQ substantially reduces the membrane cleaning frequency.

Cheong et al. [38,52] investigated the effectiveness of *Pseudomonas* sp. 1A1 to mitigate biofouling in MBRs for running times ranging from 6 to 13 days. Concerning the comparison of the times t and t’ at which the breaking points C and C’ were reached for the control and the QQ MBR, respectively (Figure 7), the time elapsing before the TMP jump was observed to increase by 180% (corresponding to an almost threefold postponement) (reference n° 1 in Table 2). When comparing the slopes corresponding to the TMP jump sections CD and C’D’ (Figure 7), the times necessary to attain the pressures of 25 kPa and 40 kPa were both seen to be delayed by approximately 3 days (reference n° 1 in Table 2). Although these results reveal that *Pseudomonas* sp. 1A1-mediated QQ does indeed have an effect on biofouling, more research is needed to unravel how *Pseudomonas* sp. 1A1 mitigates biofouling in MBRs and how its effectiveness evolves over time.

### 8.3. Effect on the Mixed Liquor Characteristics

It is important to evaluate the effect of QQ on the mixed liquor characteristics, particularly in terms of EPS and AHL amounts, since these are good indicators of biofouling.

EPS are well-known to be closely related to biofouling in MBRs since they are the “glue” that holds the biofilm cell clusters attached to the membrane [2]. In other words, a noticeable increase in the amount of EPS in the MBR is correlated with a heightened biofouling phenomenon. The total quantity of EPS in the mixed liquor can be divided into soluble microbial products (SMP) and EPS bound to the microbial flocs. In principle, these fractions need to be collected separately to be further analyzed (for more details on EPS extraction methods, see Domínguez et al. [61]). With the implementation of *Rhodococcus* sp. BH4 as a QQ bacterium, Maqbool et al. [27] determined the amount of SMP in the mixed liquor by analyzing the supernatant from an AS sample (reference n° 6 in Table 1). A 90% reduction in the SMP amount was recorded after 80 days of operation, indicating that *Rhodococcus* sp. BH4-mediated QQ had a strong effect on the EPS production. In addition, Lee et al. [26] recorded 52% and 85% reductions in the amounts of proteins and polysaccharides in the mixed liquor, respectively (reference n° 14 in Table 1). It is important to quantify this effect since it has been shown that, in some cases, the solutes and colloids in the supernatant play a more important role in biofouling than the biological pellets [62,63]. Nevertheless, it is still worth mentioning that slight reductions in the amounts of bound EPS were obtained with the application of QQ, with −32% and −5% in the loosely-bound EPS (LB-EPS) and the tightly-bound EPS, respectively [27]. Lee et al. [26] also recorded an average reduction of 17% in the amount of bound EPS in mixed liquor.

Concerning the amount of AHL in the mixed liquor, given that these signal molecules are usually produced at very low concentrations (in the range of picograms to nanograms per liter) and that they are present as a complex mixture with different compounds, an extraction procedure is necessary before the quantification [64,65]. Several quantification methods to measure the AHL concentration after their extraction have been reported to date and are summarized by Siddiqui et al. [5]. Maqbool et al. [27] extracted the AHLs from the supernatant of a broth sample then analyzed them using HPLC. A much smaller AHL concentration was observed in the QQ-MBR than in the control MBR (qualitative results). Hence, Maqbool et al. [27] came to the conclusion that the implementation of *Rhodococcus* sp. BH4 leads to a biofouling reduction via the destruction of AHLs in the mixed liquor, which is consistent with previous studies [24]. This result shows that monitoring the AHL concentration in the mixed liquor could help evaluate the progress of the QQ activity in MBRs. However, no information about the evolution of this concentration during the MBR operation is provided in the studies noted in Table 1, probably because of the very low amounts, which would make the quantification laborious.

Recently, Lee et al. [26] showed that QQ could have an effect on the floc size of the AS. In a pilot-scale MBR of 80 L, they recorded a 17% reduction in the average floc size. However, in a three-stage MBR of a total working volume of 450 L, composed of three tanks of 150 L (anoxic, aerobic and membrane tank), QQ did not lead to major differences in floc size in each tank. Thus, there is no clear trend yet about the influence of *Rhodococcus* sp. BH4-mediated QQ on the floc size of the mixed liquor.

Finally, additional research into the effect of QQ on other properties such as zeta potential, viscosity, or Sludge Volume Index (SVI) is required to provide complementary data to understand exactly how *Rhodococcus* sp. BH4 affects the mixed liquor characteristics.

The effect of *Pseudomonas* sp. 1A1 on the sludge characteristics in an MBR has only been assessed in terms of SMP. Cheong et al. [52] have reported a 60% reduction in the amount of polysaccharides, whereas the reduction in proteins was merely 6% (references n° 4 and 5 in Table 2). These results suggest that *Pseudomonas* sp. 1A1-mediated QQ targets the QS-controlled genes in charge of the production of polysaccharides in a more pronounced way. However, at the current stage and with the few elements known so far, it is still hard to unravel the effect of *Pseudomonas* sp. 1A1-mediated QQ on the mixed liquor characteristics. Nevertheless, it is worth mentioning that some studies have investigated the QQ potential of a commercial purified acylase (porcine kidney I) that is believed to have the same mode of action as the acylase from *Pseudomonas* sp. 1A1. Yeon et al. [37] have reported reductions in the protein and the polysaccharide concentrations in the mixed liquor of approximately 60% and 20%, respectively, and Jiang et al. [34] found a 20% reduction in both the protein and the polysaccharide concentrations. Thus, there is no clear trend revealing the kind of biofilm-related genes that are affected when acylase-mediated QQ is used.

Concerning the physical characteristics of the mixed liquor, the application of the purified acylase mentioned above resulted in a lower SVI, apparent viscosity and mean particle size, and a higher zeta potential, suggesting a better filterability [34]. More investigations should be carried out though, to confirm these observations and to investigate exactly how *Pseudomonas* sp. 1A1-mediated QQ affects the mixed liquor characteristics.

### 8.4. Effect on the Biodegradation Efficiencies in the MBR

The effect on the MBR performance should obviously be evaluated to make sure the implementation of *Rhodococcus* sp. BH4-mediated QQ does not impair the MBR treatment capacities. For nine of the studies considered in Table 1 (references n° 5 to 13), this was assessed in terms of Chemical Oxygen Demand (COD) removal efficiencies and resulted in very negligible variations (less than a 3% modification compared to the control MBR) [25,27,50,51]. The fact that the COD removal efficiencies remained practically unchanged indicates that the use of *Rhodococcus* sp. BH4 as QQ bacteria induces no adverse effect on the ability of the biomass to metabolize the organic matter in the MBR. The same approach was used to assess Total Kjeldahl Nitrogen (TKN) removal and resulted in less than a 5% variation compared to the control MBR, when the *Rhodococcus* sp. BH4 cells were entrapped in CEBs, microbial vessels or RMCF (data not shown) [51]. For Lee et al. [26], neither the total nitrogen removal efficiency nor the ammonia-nitrogen (NH4-N) removal efficiency was significantly affected when *Rhodococcus* sp. BH4-meadiated QQ was used in a pilot-scale MBR. Therefore, these findings confirm that *Rhodococcus* sp. BH4-mediated QQ effectively mitigates biofouling without affecting the MBR treatment performance.

The implementation of *Pseudomonas* sp. 1A1 to mitigate biofouling in an MBR resulted in COD removal efficiencies exceeding 95%, which is not significantly different compared to that of the control MBR [38,52]. Thus, *Pseudomonas* sp. 1A1 is believed to induce no effect on the degradation of organics in MBRs. However, no information is available yet in the literature concerning the nitrogen removal efficiency.

## 9. Effect of Bacterial QQ on the MBR Performance at Microscopic Scale

At microscopic scale, the effect of bacterial QQ can be highlighted by the observation and the characterization of the biocake formed on the membrane.

### 9.1. Effect on the Amount of Biofilm

Biofouling is known to result from biocake formation on the membrane surface, of which biofilm (cells + EPS) is a major component. When studying biofouling in MBRs, the observation of biocake or biofilm becomes particularly relevant to determine whether the mitigation strategy employed, *Rhodococcus* sp. BH4-mediated QQ in this case, reduces biofilm formation effectively. In this context, membranes are usually taken out at the end of operation and specimens are cut off to measure the Total Attached Biomass (TAB) and/or to conduct microscopic observations using Confocal Laser Scanning Microscopy (CLSM) to evaluate the biofilm morphology. An approximate 50% (*w*/*w*) reduction in TAB has been reported after 1.7 and 9 days of operation by Oh et al. [28] and Kim et al. [50], respectively, while Kim et al. [25] recorded a 70% reduction after only 3 days of operation, which was in accordance with the CLSM observations (references n° 1, 5 and 13 in Table 1). Lee et al. [26] recorded the most pronounced reduction in TAB with almost 90% after 14 days of operation (reference n° 14 in Table 1). These findings clearly highlight the effectiveness of *Rhodococcus* sp. BH4-mediated QQ in reducing the amount of biofilm. However, complementary quantitative data on the biofilm thickness, porosity, etc., would be pertinent to identify possible structural modifications induced by *Rhodococcus* sp. BH4-mediated QQ.

Little is known about the effect of *Pseudomonas* sp. 1A1-mediated QQ at the microscopic scale since only two studies on that subject have been published to date [38,52]. However, Cheong et al. [52] obtained a 60% reduction in the TAB on the membrane when *Pseudomonas* sp. 1A1 bacteria-encapsulating CMV was used in the MBR (reference n° 2 in Table 2).

### 9.2. Effect on the Biofilm Composition

The biofilm composition is usually discussed in terms of EPS amounts. First, the biocake is detached from the used membrane by placing it in a water tank equipped with an air-scouring system and/or with sonication followed by an air-scouring system [25]. Finally, EPS are extracted from the biocake in suspension using the Cation Exchange Resin (CER) method developed by Jahn and Nielsen [66].

When *Rhodococcus* sp. BH4 was introduced into lab-scale MBRs to assess its QQ potential, Kim et al. [25] reported an 80% reduction in the total amount of EPS in the biocake after only 3 days of operation (reference n° 5 in Table 1). Lee et al. [26] obtained a 20% reduction in the amount of proteins, whereas the amount of polysaccharides remained practically unchanged (reference n° 14 in Table 1). On the other hand, the amounts of proteins and polysaccharides in the biocake were reduced by approximately 50% and 90% after 32 days of operation Kim et al. [50] (reference n° 13 in Table 1). These findings support the assumption that *Rhodococcus* sp. BH4-mediated QQ mitigates biofouling by decreasing the EPS production in the biofilm.

Concerning *Pseudomonas* sp. 1A1, the composition of biofilm, was reduced of about 80% and 40% in proteins and polysaccharides, respectively (reference n ° 4 in Table 2).

## 10. Effect of the Operating Conditions on the Effectiveness of Bacterial QQ

It is of great interest to investigate the effects of the operating conditions on the effectiveness of bacterial QQ, in order to identify the optimal conditions that offer the most substantial mitigation of biofouling.

### 10.1. Effect of the Initial Quantity of QQ Bacteria Inserted into the MBR

The quantity of *Rhodococcus* sp. BH4 initially inserted into the entrapping element in the MBR using PE microbial vessels has been studied for its potential effect on *Rhodococcus* sp. BH4-mediated QQ [40]. Oh et al. [40] reported that 13 days were required to reach the TMP of 30 kPa in a lab-scale MBR containing two PE microbial vessels packed with 8.9 g BH4/m^2^ of the vessel surface area, whereas it took 28 days to reach the same TMP in a lab-scale MBR containing two vessels packed with 17.8 g BH4/m^2^ of the vessel surface area (data not shown). A similar study has been reported using beads entrapping *Rhodococcus* sp. BH4 [51]. Two kinds of beads were prepared: with 1.5 mg BH4/cm^3^ of bead volume (beads I) and with 7.5 mg BH4/cm^3^ of bead volume (beads II). The two beads were placed in continuous lab-scale MBRs under the same operating conditions (references n° 7 and 8 in Table 1). Beads I reduced the TMP by 93% (reference n° 8 in Table 1), whereas beads II achieved almost 100% (reference n° 8 in Table 1). This result is thus consistent with the previous study with the microbial vessels, i.e., the greater quantity gave rise to a more significant delay of biofouling whatever the QQ device. However, the examination of a wider range of initial quantities of *Rhodococcus* sp. BH4 would be of great interest to determine an optimal quantity of cells to be introduced.

Cheong et al. [52] also experimented this effect by inserting two different initial quantities of *Pseudomonas* sp. 1A1 into the CMV: 26.5 and 70.3 mg biomass/cm^3^-lumen (corresponding to 266.4 and 706.8 mg biomass/L reactor respectively), both under the inner flow feeding mode. However, the permeate flux was not maintained constant for both initial quantities, which makes the results difficult to compare. Therefore, it could be interesting to evaluate the effect of the initial quantity on the QQ effectiveness, all other operating conditions the same.

Finally, assessments of effect of the filtration mode (continuous, relaxation, backwash), the membrane design (material, geometry, configuration), the entrapping method, or the aeration rate, could be particularly relevant in an attempt to optimize application of *Pseudomonas* sp. 1A1-mediated QQ.

The operating conditions mentioned here are the only ones that have been investigated to date. However, other parameters such as the aeration rate, the total working volume, and the recirculation rate in a side-stream MBR, should be examined for their potential influence on the effectiveness of *Pseudomonas* sp. 1A1-mediated QQ.

### 10.2. Effect of the Entrapping Method

The mass transfer question has been further moved ahead by an analysis of the effect of the entrapping method on the *Rhodococcus* sp. BH4 efficiency in mitigating biofouling. The three entrapping methods mentioned above (microbial vessels, beads and RMCF) were compared under the same operating conditions with the same quantity of *Rhodococcus* sp. BH4 [51] (references n° 10 to 12 in Table 1). After 1 day of operation, beads were found to be the most efficient, with a reduction of 90% in the TMP level (reference n° 10 in Table 1) and the microbial vessel was the least efficient to reduce biofouling (reference n° 11 Table 1).

It is noteworthy that the TMP jump obtained with the beads seems to be the most pronounced, with only a 14% fall in the TMP at the end of operation. This observation still remains unexplained, though Köse-Mutlu et al. [51] proposed a global assessment of the BH4 QQ effectiveness based on the ratio between the areas under the TMP curves. Consequently, based on these ratios, it was concluded that the more mobile the QQ device is, the more efficient is the biofouling mitigation via *Rhodococcus* sp. BH4-mediated QQ. This can be attributed to the higher frequency of contacts between QQ media and AHL molecules promoted by the movement of QQ media. Another reason would be that the free movement of the beads induces collisions between beads and membrane, resulting in physical cleaning in addition to the QQ effect, which thus enhances biofouling mitigation. This is consistent with previous studies demonstrating that vacant beads (with no *Rhodococcus* sp. BH4) or other inert particles also had the ability to reduce biofouling through a physical washing action that considerably delayed the time before the TMP jump was reached [25,67].

### 10.3. Effect of the Materials Used in the QQ Device

The effect of the microbial vessel material on the QQ effectiveness has also been investigated [40]. Four kinds of microbial vessels were designed, with different materials, pore sizes, surface areas and inner volumes. Each of the four vessels was packed with the same quantity of QQ bacteria (30 mg BH4/vessel). The influence of the microbial vessel design on the QQ effectiveness was evaluated for approximately 40 days of operation in MBRs with a working volume of 20 L. At certain times, the vessels were removed from the MBR to measure their QQ activity based on the degradation rate of C8-HSL over 45 days. The modified polyethersulfone membrane (PESM), which had the largest inner volume (0.98 mL), was found to offer the highest QQ activity, with around 60% after 45 days. In comparison, the PE membrane and the two PVDF membranes with smaller inner volumes (0.20, 0.31 and 0.29 mL, respectively) only resulted in 20%–45% after 45 days. This was attributed to the fact that the largest volume offered more space for the *Rhodococcus* sp. BH4 growth.

### 10.4. Effect of the Location of the Microbial Vessel in the MBR

The effect of the specific location of the microbial vessel inside the MBR is another important factor that has been investigated. Jahangir et al. [49] designed a side-stream MBR (or external submerged MBR) with a total working volume of 2.8 L consisting of a bioreactor tank (2 L) and a membrane tank (0.8 L), with continuous recirculation between the two tanks (7.5 to 30 mL/min). The operating conditions are noted in Table 1 (references n° 2 and 3). In order to highlight the significance of the location of *Rhodococcus* sp. BH4 strain in the MBR, the microbial vessel was placed either in the bioreactor tank or in the membrane tank to carry out a comparative study in terms of TMP level. An overall enhancement of the *Rhodococcus* sp. BH4-mediated QQ activity was obtained when the microbial vessel was near the membrane, with a 50% reduction in the TMP after 1 day of operation and about an 80% reduction at the end of the operation. The corresponding reductions were approximately 20% and 35% when the microbial vessel was in the bioreactor. The time required to reach a TMP of 25 kPa was extended by 160% when the vessel was in the membrane tank, whereas, it was delayed by only 110% when the vessel was in the bioreactor. Therefore, the closer the microbial vessel is to the membrane, the greater is the capacity of *Rhodococcus* sp. BH4 to mitigate biofouling through QQ activity. This finding is of primary importance because it gives essential information about the AHLs that *Rhodococcus* sp. BH4-mediated QQ targets. In this case, it is assumed that the AHLs in the biofilm on the membrane surface play a more significant role in biofouling than the AHLs from the mixed liquor. In addition, the importance of the question of mass transfer is raised again with this experiment since the nearer the QQ enzyme (intracellular AHL-lactonase) was to its substrate (AHLs from biofilm), the more pronounced was the biofouling mitigation in the QQ MBR.

### 10.5. Effect of the Recirculation Rate in a Side-Stream MBR

With the same side-stream MBR as above (or external submerged MBR), Jahangir et al. [49] considered the *Rhodococcus* sp. BH4-mediated QQ effectiveness as a function of the recirculation rate between the two tanks. For that purpose, the recirculation rate was set to 7.5, 15 and 30 mL/min and the effect on QQ effectiveness was assessed in terms of TMP. It was found that the QQ effectiveness increased with the recirculation rate, for both of the microbial vessel positions (data not shown). Besides, the QQ effectiveness appeared to be much more enhanced by an increased recirculation rate when the microbial vessel was in the membrane tank, than when it was in the bioreactor. These results again reinforce the significance of molecules transport, since they indicate that a greater recirculation rate promotes the transport of AHLs from the biofilm on the membrane surface so that they can be degraded inside the microbial vessel.

### 10.6. Effect of Coupled Physical Cleaning Methods

Another study focused on the coupling of *Rhodococcus* sp. BH4-mediated QQ with two physical cleaning modes: relaxation and air backpulse [30]. The former consisted in a short break (1 min) after 19 or 29 min of filtration, while the latter consisted in injecting compressed air into the lumen side of the membrane for 20 s. In MBRs equipped with *Rhodococcus* sp. BH4-entrapping microbial vessels, TMP monitoring over approximately 20 days of operation revealed that the QQ+air backpulse combination gave rise to a more significant biofouling reduction than QQ+relaxation (data not shown).

### 10.7. Effect of the Permeate Flux

The effect of the permeate flux on the *Rhodococcus* sp. BH4-mediated QQ effectiveness was also investigated with beads as the entrapping method. Köse-Mutlu et al. [51] reported that an increase in the permeate flux from 30 to 50 L/m^2^·h severely impacted the *Rhodococcus* sp. BH4-mediated QQ effectiveness in terms of TMP reduction at the end of operation (−97% at 30 L/m^2^·h versus −14% at 50 L/m^2^·h). This can be explained by the fact that the formation of biocake is facilitated by the driving forces created with a high permeate flux. Another possible explanation is that the transport of AHLs from the biocake to the entrapped *Rhodococcus* sp. BH4 cells can be strongly counteracted by a high permeate flux.

In addition to the previous operating conditions, it could be of interest to investigate the effects of other parameters such as the aeration rate or the total working volume on the effectiveness of the *Rhodococcus* sp. BH4-mediated QQ with a view to identifying the optimal value for QQ efficiency.

From evaluations of the permeate flux using *Pseudomonas* sp. 1A1, it has been reported that a decrease in the permeate flux from 30 L/m^2^·h to 25 L/m^2^·h gives rise to slower biofouling (references n° 2 and 3 in Table 2). As presented in Table 2, the *Pseudomonas* sp. 1A1 effect on biofouling mitigation seems to be unchanged, since the TMP was reduced by approximately 80% at the end of the MBR operation with the addition of QQ, under both of the permeate fluxes. Thus, modification of the permeate flux seemed to have no specific effect on the QQ effectiveness, but did have an effect on the biofouling kinetics in both the control and the QQ MBRs.

### 10.8. Effect of the Feeding Mode in Case of CMV for Entrapping Pseudomonas sp. 1A1

When a CMV was used as an entrapping device, the feeding mode made a significant difference in the performance of the QQ MBR [52]. The CMV was composed of a central lumen surrounded by six other lumens in a circle, into each of which *Pseudomonas* sp. 1A1 was inserted (Figure 4c). The experiments carried out with 266.4 mg/L of *Pseudomonas* sp. 1A1 initially inserted into the MBR and with a permeate flux of 35 L/m^2^·h, showed that the inner flow feeding mode (i.e., MBR fed though the central lumen of the submerged CMV) resulted in an overall enhancement of the QQ effectiveness compared to the normal feeding mode (directly in the MBR tank) (references n° 4 and 5 in Table 2). The TMP rise-up corresponding to the inner flow feeding mode was substantially delayed compared with that corresponding to the normal feeding flow, the TMP being approximately 60% at the end of the operation for the inner mode, versus 25% lower for the normal mode. In addition, the effect on the mixed liquor and the biocake composition in terms of EPS was more pronounced when the inner flow feeding mode was used (references n° 4 and 5 in Table 2). Under the normal mode, *Pseudomonas* sp. 1A1 lost most of its QQ effectiveness over time with, with a 25% reduction in the TMP at the end of the operation, versus about 50% at the end of the first cycle (reference n° 5 in Table 2), whereas the *Pseudomonas* sp. 1A1 activity remained practically unchanged under the inner mode (reference n° 4 in Table 2).

In conclusion, the inner flow feeding mode gave rise to enhanced QQ activity of *Pseudomonas* sp. 1A1. Cheong et al. [52] attributed these observations to the higher viability of the encapsulated cells that occurred with relatively fresher feed by the inner flow feeding mode. In fact, the inner feeding is believed to enhance the mass transfer of nutrients from the feed to the *Pseudomonas* sp. 1A1 encapsulated cells, promoting the growth of the bacteria as well as their acylase production. However, it can also be assumed that the inner flow feeding mode would foster the transport of acylases by driving them from the CMV lumens to the mixed liquor, thereby increasing their probability of encountering and degrading the signal molecules of AHL molecules. The latter hypothesis would need to be confirmed by a comparative study of acylase mass transfer through the CMV in the two feeding modes.

## 11. Discussion and Concluding Remarks

The results that have been presented in this review for the two QQ strains considered (*Rhodococcus* sp. BH4 and *Pseudomonas* sp. 1A1) highlight some interesting features of bacterial QQ for membrane biofouling mitigation in MBRs. The mode of action of each strain has been discussed through the nature and the localization of the QQ capacity and its effect on the MBR at both the macroscopic and the microscopic scales. The QQ effectiveness has been further discussed as a function of the operating conditions.

Both the strains considered showed a great potential to mitigate biofouling. However, even though the QQ bacteria considered are two particular cases that have been investigated so far, the confrontation of their assumed modes of action has helped underline some gaps in research on the application of QQ for membrane biofouling control in MBRs. Below, we attempt to summarize the main interrogations that remain unsolved to date.

- When studying the effect of the two bacteria on the progressive biofouling stage, it appeared that *Pseudomonas* sp. 1A1 had a less pronounced effect in terms of TMP reduction. Yet, it has been assumed that the effectiveness of *Pseudomonas* sp. 1A1 in mitigating membrane biofouling could be expected to be more significant, given that the QQ enzyme that it produces (acylase) is extracellular. Hence, a comparative study with *Rhodococcus* sp. BH4 under the exact same conditions could help identify the possible differences between these two QQ bacteria.
- In this work, the presentation of the QQ activity localization and the entrapping methods for both the QQ bacteria considered helped underline that the transport of the main molecules involved in the QQ process (AHLs as substrates and lactonases or acylases as QQ enzymes) is not completely understood yet, although some assumptions have been made in that direction. Hence, an in-depth characterization of the transport of these molecules in the MBR should be carried out, given the MBR hydrodynamics and the variety of methods existing for QQ bacteria entrapment.
- The analysis of the bacterial QQ effect on the progressive biofouling stage has given some interesting clues about the time needed for the QQ activity to become significant in the MBRs. It appears that the QQ activity takes place in the early phase of the MBR operation for both *Rhodococcus* sp. BH4 and *Pseudomonas* sp. 1A1. Thus, it could be of great interest for both of these QQ strains to investigate which, of QQ enzyme production or QQ enzyme transport, is the limiting step in the QQ process for biofouling mitigation in MBRs.
- In all the studies considered in this review, the TMP jump was successfully delayed with the application of bacterial QQ over the run times investigated. However, bacterial QQ does not completely prevent biofouling; it simply postpones its occurrence. One of the hypotheses to explain this observation could be that other kinds of QS-controlled biofouling become prevalent in the MBRs. Thus, the relationship between biofouling and other kinds of autoinducers present in the MBR (i.e., AIPs and AI-2s) should be investigated, and might lead to a more efficient QQ strategy to mitigate biofouling.

We discussed QQ for membrane biofouling mitigation in MBRs based on the detailed functioning of two particular QQ bacteria: *Rhodococcus* sp. BH4 and *Pseudomonas* sp. 1A1. The information gathered for that purpose led to the conclusion that bacterial QQ appears to be a promising strategy to fight the main obstacle that currently hinders the spread of MBRs: biofouling. The application of bacterial QQ to MBRs gave very good results that could be quantified at both the macroscopic and the microscopic scales. Besides, QQ was found to induce significant energy savings in MBRs. As an example, about 30% of reduction in the specific aeration energy demand was obtained in a lab-scale QQ-MBR [30], and the filtration energy consumption was reduced by approximately 50% in a lab-scale QQ-MBR equipped with a chlorine-based backwashing system [68]. The bacterial QQ was also found to be effective to reduce biofouling in a pilot-scale MBR fed with real WW. Moreover, Lee et al. [26] recently demonstrated in a pilot-scale MBR that the application of QQ could reduce by approximately 60% the biofouling related energy consumption. Thus, the fact that the application of bacterial QQ leads to a significant reduction in both the energy consumption and the operating costs is an additional strong advantage showing that this is a viable method that could be used as the sole anti-biofouling strategy or, at least in the near future, as a complement to physical cleaning methods.

On the other hand, it appeared that the QQ effectiveness in mitigating biofouling depends on the MBR operating conditions. Consequently, it has been reported that, the greater the initial quantity of QQ bacteria (*Pseudomonas* sp. 1A1 or *Rhodococcus* sp. BH4) inserted in the MBR, the more efficient bacterial QQ can be to reduce membrane biofouling. Concerning the different entrapping methods, it has been demonstrated that the mobile entrapping elements (RMCF, CEBs and macrocapsules) give rise to enhanced QQ effectiveness in comparison to the stationary microbial vessel, probably because of the shear forces they create, which would promote the transport of the AHLs and/or induce a direct physical cleaning effect of the biocake on the membrane. With the use of the microbial vessel, it has been shown that QQ is more effective to reduce biofouling when the vessel is closer to the membrane, which indicates that AHLs from the biocake play an important role in the QQ process. Also, a higher recirculation rate in a side-stream MBR has been found to result in heightened QQ effectiveness. Finally, the permeate flux has been identified as having a negative influence on the effectiveness of both the QQ bacteria (*Pseudomonas* sp. 1A1 and *Rhodococcus* sp. BH4) for the mitigation of membrane biofouling in MBRs.

Therefore, we can conclude that great efforts have been made to optimize the bacterial QQ process, in particular by investigating the possible relationships between QQ effectiveness and the MBR operating conditions. Nevertheless, some crucial information is still missing and future research should focus on the few gaps revealed in order to make the application of bacterial QQ conceivable for membrane biofouling mitigation in MBRs in the near future.

## Figures and Tables

**Table 1:** Effect of *Rhodococcus* sp. BH4-mediated Quorum Quenching on membrane biofouling mitigation in MBRs inoculated with activated sludge (AS), under continuous mode and using hollow fibers (HF) or flat sheet (FS) membrane as the filtration module.

QQ MBR Design									MBR Operation										Resulting QQ Effect Expressed in% of the Control MBR												Ref.	N°
Reactor		Membrane					*Rhodococcus* sp. BH4																									
Geometry	Working Volume (L)	Nature	Geometry	Configuration	Area (cm^2^)	Pore Size (µm)	Entrapping Method	Inserted Quantity of BH4 Cells in the Reactor (mg/L)	F/M Ratio	MLSS (mg/L)	HRT (h)	SRT (d)	Permeate Flux (L/m^2^ h)	Air Supply (m^3^/h)	Filtration/Relaxation	Run Time (days)	Number of Cycles		TMP			Time for TMP to Reach			TAB in Biofilm	SMP in Mixed Liquor		EPS in Biofilm		COD removal Efficiency		
																	For Control MBR	For QQ MBR	After 1 Day of Operation	At the End of the 1st cycle	At the End of Operation	The Breaking Point	25 kPa	40 kPa		Proteins	Polysaccharides	Proteins	Polysaccharides			
**Synthetic wastewater**																																
○	1.2	PVDF	HF	Submerged	86	0.04	PE microbial vessels (2/reactor)	*450*	n.a.	4500–5000	12	40	18	n.a.	-	3.75	2	2	*−68%*	*−51%*	*−61%*	*-*	*+148%*	*-*	*−*49 w % after 1.7 day	-	-	-	-	-	[28]	1
○	2.8 (2 L bioreactor + 0.8 L membrane tank)	PVDF	HF	Side-stream (external submerged)	120	0.04	PE microbial vessel (1 in the bioreactor)	*128.6*	0.22	n.a.	12	50	30	0.09 in the bioreactor and 0.06 in the membrane tank	60 min/1 min	1.4	1	1	*−23%*	*−35%*	*−35%*	*-*	*+110%*	*-*	-	-	-	-	-	-	[49]	2
○	2.8 (2 L bioreactor + 0.8 L membrane tank)	PVDF	HF	Side-stream (external submerged)	120	0.04	PE microbial vessel (1 in the membrane tank)	*128.6*	0.22	n.a.	12	50	30	0.09 in the bioreactor and 0.06 in the membrane tank	60 min/1 min	1.75	1	1	*−50%*	*−77%*	*−77%*	*+240%*	*+160%*	*-*	-	-	-	-	-	-	[49]	3
n.a.	1.2	PVDF	HF	Submerged	86	0.04	PE microbial vessels (2/reactor)	*167*	0.17–0.2	7000–7500	10	40	35	n.a.	-	5	1	1	*−18%*	*−68%*	*−68%*	*+300%*	*+216% (+2.8 day)*	*+205%(+3.1 day)*	-	-	-	-	-	-	[40]	4
○	1.6	PVDF	HF	Submerged	13.4	0.04	CEBs (40/reactor)	*~8*	n.a.	12,500–13,000	5.3	25	28.7	n.a.	-	17	4	1	*−10%*	*−93%*	*−94%*	*+672%*	*+548% (+14.3 day)*	*+504% (+14.2 day)*	*−*69 w % after 3.1 days	-	-	*−*81% after 3.1 days *		*+0.2%*	[25]	5
□	35	PVDF	HF	Submerged	7000	0.1	CEBs (1860/reactor)	n.c.	n.a.	10,000	4	20	15	1.08	8 min/2 min	90	7	1	*−24%*	*−88%*	*−37%*	*+550%*	*+724%*	*-*	-	*−*89% after 80 days		-	-	*−0.3%*	[27]	6
○	5	n.a.	HF	Submerged	100	n.a.	CEBs	n.c.	n.a.	12,000–13,000	13	30	20	n.a.	-	3.8	1	1	*−85%*	*−93%*	*−93%*	*-*	*-*	*-*	-	-	-	-	-	*+1.6%*	[51]	7
○	5	n.a.	HF	Submerged	100	n.a.	CEBs	n.c.	n.a.	12,000–13,000	13	30	20	n.a.	-	2.4	1	1	*−98%*	*−99.6%*	*−99.6%*	*-*	*-*	*-*	-	-	-	-	-	*+2.5%*	[51]	8
○	5	n.a.	HF	Submerged	100	n.a.	CEBs	n.c.	n.a.	12,000–13,000	13	30	30	n.a.	-	3.5	1	1	*−95%*	*−97%*	*−97%*	*-*	*-*	*-*	-	-	-	-	-	*~0%*	[51]	9
○	5	n.a.	HF	Submerged	100	n.a.	CEBs	n.c.	n.a.	12,000–13,000	13	30	50	n.a.	-	1.7	1	1	*−90%*	*−14%*	*−14%*	*-*	*+295%*	*-*	-	-	-	-	-	*~0%*	[51]	10
○	5	n.a.	HF	Submerged	100	n.a.	PVDF microbial vessel (1/reactor)	n.c.	n.a.	12,000–13,000	13	30	50	n.a.	-	1.9	1	1	*−49%*	*−40%*	*−40%*	*-*	*+138%*	*-*	-	-	-	-	-	*+2%*	[51]	11
○	5	n.a.	HF	Submerged	100	n.a.	RMCF	n.c.	n.a.	12,000–13,000	13	30	50	n.a.	-	1.9	1	1	*−59%*	*−63%*	*−63%*	*-*	*-*	*-*	-	-	-	-	-	*~0%*	[51]	12
**Real wastewater**																																
□	2.5	PVDF	HF	Submerged	155	0.04	Macrocapsules (500/reactor)	n.c.	0.1–0.2	5300–5700	8	30	30	0.06	-	22	3	1	*−23%*	*−87%*	*−21%*	*+1135%*	*+750% (+ 19 day)*	*+667% (+19.7 day)*	*−*48 w % after a 9 days cycle	-	-	*−*53%	*−*88%	*+0.5%*	[50]	13
n.a.	80	C-PVC	FS	Submerged	9000	0.4	QQ beads (~19000/reactor)	~4000	n.a.	10,000–13,000	5.2	25	20	0.27	10 min/ 2 min	14	2	1	n.c.	*−83%*	*−81%*	*+590%*	*+470%*	-	*−*86% after 14 days	*−*52%	*−*85%	*−*21%	-	-	[26]	14

○: Cylindrical; □: Parallelepiped; Numbers: Taken from the literature; *Numbers*: Calculated with the data provided in the literature; n.a./-: Not available data; n.c.: Not calculable value with the data provided.

**Table 2:** Effect of *Pseudomonas* sp. 1A1-mediated Quorum Quenching on membrane biofouling mitigation in MBRs inoculated with AS, under continuous mode and using submerged polyvinylidene fluoride (PVDF) hollow fibers as the filtration module.

MBR Design							MBR Operation								Resulting QQ Effect Expressed in% of the Control MBR											Ref.	N°
Reactor		Membrane			*Pseudomonas* sp. 1A1																						
Geometry	Working Volume (L)	Configuration	Area (cm^2^)	Pore Size (µm)	Entrapping Method	Inserted Quantity of 1A1 Cells in the Reactor (mg/L)	F/M Ratio	MLSS (mg/L)	HRT (h)	SRT (d)	Permeate Flux (L/m^2^ h)	Run Time (days)	Number of Cycles		TMP			Time for TMP to Reach			TAB in Biofilm	SMP in Mixed Liquor		EPS in Biofilm			
													For Control MBR	For QQ MBR	After 1 Day of Operation	At the End of the 1st Cycle	At the End of Operation	The Breaking Point	25 kPa	40 kPa		Proteins	Polysaccharides	Proteins	Polysaccharides		
○	2.5	Submerged	155.2	0.04	PE vessels (4/reactor)	*192*	0.20–0.22	7600–8000	8	30	25	7.8	2	2	*−15%*	*−71%*	*−79%*	*+180%*	*+134% (+2*.*6 day)*	*+150% (+3*.*1 day)*	*-*	*-*	*-*	*-*	*-*	[38]	1
○	3.0	Submerged	210	0.04	CMV under inner flow feeding mode (1/reactor)	*706*.*8*	0.12–0.21	11,000–13,000	6	60	30	2.1	1	1	*−15%*	*−77%*	*−77%*	*-*	*-*	*-*	*−63 w%*	*-*	*-*	*-*	*-*	[52]	2
○	3.0	Submerged	210	0.04	CMV under inner flow feeding mode (1/reactor)	*706*.*8*	0.12–0.21	11,000–13,000	6	60	25	9.4	1	1	*−22%*	*−76%*	*−76%*	*-*	*-*	*-*	*-*	*-*	*-*	*-*	*-*	[52]	3
○	3.0	Submerged	210	0.04	CMV under inner flow feeding mode (1/reactor)	*266*.*4*	0.12–0.21	11,000–13,000	6	60	35	6	2	2	*−25%*	*−60%*	*−56%*	*-*	*-*	*-*	*-*	*−6%*	*−62%*	*−77%*	*−37%*	[52]	4
○	3.0	Submerged	210	0.04	CMV under normal feeding mode (1/reactor)	*266*.*4*	0.12–0.21	11,000–13,000	6	60	35	6	2	2	*−5%*	*−44%*	*−25%*	*-*	*−3%*	*-*	*-*	*−2%*	*−51%*	*−31%*	*−36%*	[52]	5

○: Cylindrical; Numbers: Taken from the literature; *Numbers*: Calculated with the data provided in the literature; n.a./-: Not available data; n.c.: Not calculable value with the data provided.

## Supplementary Materials

The supplementary materials are available online at http://www.mdpi.com/2077-0375/6/4/52/s1. Figure S1: AHL-mediated QS in Gram-negative bacteria (**1**) Synthesis of the LuxI protein resulting from *luxI* gene transcription; (**2**) Synthesis of the LuxR receptor resulting from *luxR* gene transcription; (**3**) Biosynthesis of AHL catalyzed by the LuxI protein; (**4**) Diffusion of AHL out of the cell and increase of the extracellular concentration; (**5**) Increase of the intracellular concentration of AHL; (**6**) Formation of the LuxR-AHL complex; (**7**) Activation of the transcription of the target genes (biofilm-related genes) and *luxI* gene transcription (positive autoregulatory loop).

## Nomenclature

AHL	*N*-acyl-l-homoserine lactone
AI	Autoinducer
AS	Activated Sludge
CEB	Cell Entrapping Bead
CLSM	Confocal Laser Scanning Microscope
CMV	Ceramic Microbial Vessel
COD	Chemical Oxygen Demand
EPS	Extracellular Polymeric Substances (or Exopolysaccharides)
F/M	Food-to-Mass ratio
HF	Hollow Fiber
HPLC	High Performance Liquid Chromatography
HRT	Hydraulic Retention Time
HSL	Homoserine Lactone
LB (biofilm)	Loosely Bound
LB (medium)	Luria-Bertani
MBR	Membrane Bioreactor
MEC	Magnetic Enzyme Carrier
MLSS	Mixed Liquor Suspended Solids
PBE	Piper Betle Extract
PE	Polyethylene
PESM	Modified Poly Ether Sulfone
PVDF	Polyvinylidene fluoride
QQ	Quorum Quenching
QS	Quorum Sensing
QSI	Quorum Sensing Inhibitors
SMP	Soluble Microbial Product
SRT	Solid Retention Time
SVI	Sludge Volume Index
TAB	Total Attached Biomass
TB	Tightly Bound
TMP	Transmembrane Pressure
TKN	Total Kjeldahl Nitrogen
WW	Wastewater
WWT	Wastewater Treatment
