# Supplementary material for: Process-Oriented Review of Bacterial Quorum Quenching for Membrane Biofouling Mitigation in Membrane Bioreactors (MBRs)
Source: Membranes (Basel). 2016 Dec 13;6(4):52. doi: 10.3390/membranes6040052 (PMC5192408; doi:10.3390/membranes6040052)
Supplement: Supplementary file 1 [file membranes-06-00052-s001.pdf]

Supplementary Materials: Process-Oriented Review of Bacterial Quorum Quenching for Membrane Biofouling Mitigation in Membrane Bioreactors (MBRs)

Naila Bouayed, Nicolas Dietrich, Christine Lafforgue, Chung-Hak Lee and Christelle Guigui

Figure S1 presents a schematic illustration of the AHL-mediated QS in Gram-negative bacteria. The AHL-mediated QS is based on the *luxI-luxR* regulatory system that was first defined by Fuqua et al. [1]. The *luxI* gene codes for the production of the LuxI protein that catalyzes the AHL biosynthesis (steps (1) and (3) in Figure 2). Once the AHLs are produced, they accumulate both in the intracellular and the extracellular environment because of their small molecular weights, which range from approximately 170 to 300 g·mol^{-1}. At high cell density, the extracellular concentration of AHLs increases until it reaches a threshold, beyond which the AHLs bind intracellularly to the receptor protein encoded by the *luxR* gene to form a LuxR-AHL complex (step (6) in Figure 2). This complex then activates the transcription of several target genes, including the *luxI* gene, which creates a positive autoregulatory loop (step (7) in Figure 2). For more details on QS mechanisms, the reader is invited to refer to Miller and Bassler [2], Parsek and Greenberg [3], Waters and Bassler [4], Whitehead et al. [5] and Williams [6].

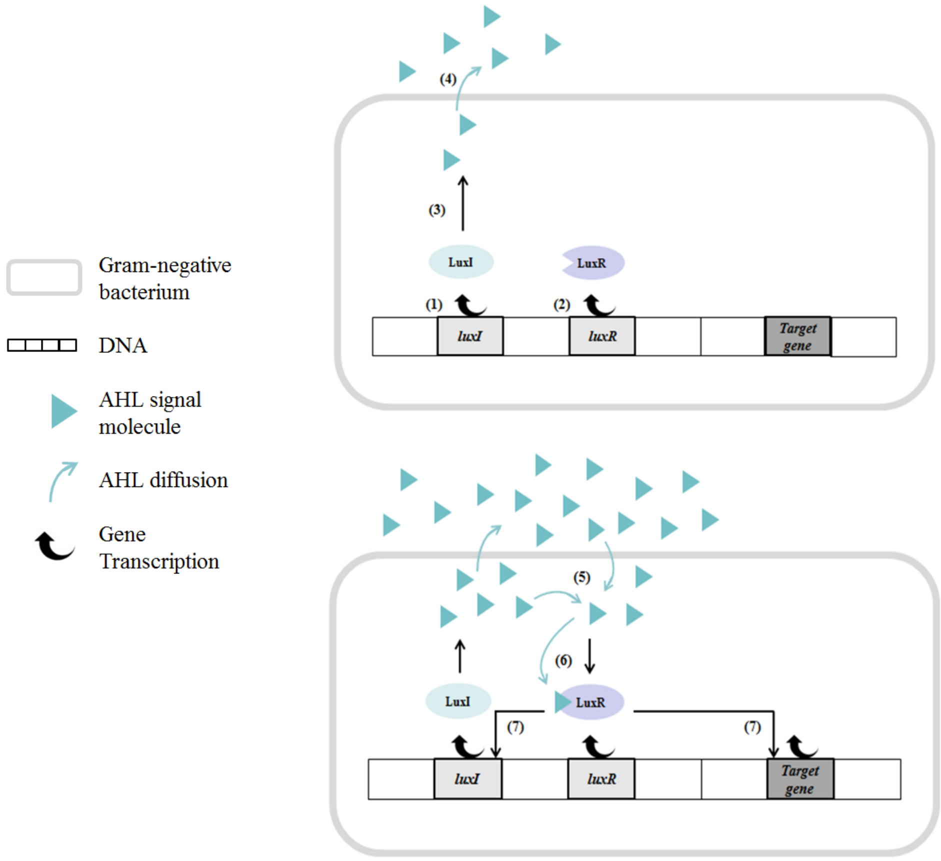

Figure S1. AHL-mediated QS in Gram-negative bacteria (**1**) Synthesis of the LuxI protein resulting from *luxI* gene transcription; (**2**) Synthesis of the LuxR receptor resulting from *luxR* gene transcription; (**3**) Biosynthesis of AHL catalyzed by the LuxI protein; (**4**) Diffusion of AHL out of the cell and increase of the extracellular concentration; (**5**) Increase of the intracellular concentration of AHL; (**6**) Formation of the LuxR-AHL complex; (**7**) Activation of the transcription of the target genes (biofilm-related genes) and *luxI* gene transcription (positive autoregulatory loop).

References

1. Fuqua, W.C.; Winans, S.C.; Greenberg, E.P. Quorum sensing in bacteria: the LuxR-LuxI family of cell density-responsive transcriptional regulators. *J. Bacteriol.* **1994**, *176*, 269–275.
2. Miller, M.B.; Bassler, B.L. Quorum Sensing in Bacteria. *Annu. Rev. Microbiol.* **2001**, *55*, 165–199.
3. Parsek, M.R.; Greenberg, E.P. Acyl-homoserine lactone quorum sensing in Gram-negative bacteria: A signaling mechanism involved in associations with higher organisms. *Proc. Natl. Acad. Sci. USA* **2000**, *97*, 8789–8793.
4. Waters, C.M.; Bassler, B.L. Quorum sensing: Cell-to-cell communication in bacteria. *Annu. Rev. Cell Dev. Biol.* **2005**, *21*, 319–346.
5. Whitehead, N.A.; Barnard, A.M.L.; Slater, H.; Simpson, N.J.L.; Salmond, G.P.C. Quorum-sensing in Gram-negative bacteria. *FEMS Microbiol. Rev.* **2001**, *25*, 365–404.
6. Williams, P. Quorum sensing, communication and cross-kingdom signalling in the bacterial world. *Microbiology* **2007**, *153*, 3923–3938.
